# The beneficial effects of intradialytic parenteral nutrition in hemodialysis patients with protein energy wasting: a prospective randomized controlled trial

**DOI:** 10.1038/s41598-022-08726-8

**Published:** 2022-03-16

**Authors:** Piyawan Kittiskulnam, Athiphat Banjongjit, Kamonchanok Metta, Khajohn Tiranathanagul, Yingyos Avihingsanon, Kearkiat Praditpornsilpa, Kriang Tungsanga, Somchai Eiam-Ong

**Affiliations:** 1 Division of Internal Medicine-Nephrology, Department of Medicine, Faculty of Medicine, Chulalongkorn University and King Chulalongkorn Memorial Hospital, Thai Red Cross Society, Bangkok, Thailand; 2 Division of Nephrology, Department of Medicine, Faculty of Medicine, Chulalongkorn University, Bangkok, 10330 Thailand; 3 Special Task Force for Activating Research in Renal Nutrition (Renal Nutrition Research Group), Office of Research Affairs, Chulalongkorn University, Bangkok, Thailand

**Keywords:** Medical research, Nephrology

## Abstract

In hemodialysis (HD) patients, protein-energy wasting (PEW) is highly prevalent and firstly treated with oral nutritional supplements (ONS). The extent to which intradialytic parenteral nutrition (IDPN) contributes to improve PEW status in HD patients intolerable to ONS remains unclear. Maintenance PEW HD patients being unable to tolerate ONS adverse effects, and having spontaneous energy and protein intake of ≥ 20 kcal/kg/day and ≥ 0.8 g/kg/day, respectively were randomly assigned 1:1 into IDPN and control groups. In IDPN group, most concentrated 3-in-1, fish-oil based parenteral nutrition was infused during HD for 3 months. The control group received intensive dietary counselling once weekly for 3 months. Both groups were then followed for additional 3 months after intervention. A total of 38 patients were randomized (mean age 67.6 years). After 3 months, serum albumin was significantly higher in the IDPN (n = 18) compared with control group (from 3.5 ± 0.3 to 3.8 ± 0.2 vs from 3.6 ± 0.3 to 3.5 ± 0.3 g/dL, respectively, *p* = 0.01). Spontaneous dietary intake (*p* = 0.04), body weight (*p* = 0.01), and malnutrition inflammation score (MIS, *p* = 0.01) were improved in the IDPN, but not in the control group. Muscle mass, strength, serum prealbumin, interleukin-6, high sensitivity-c reactive protein, and acylated ghrelin were not significantly different but leptin levels increased in the control group after 3 months (*p* = 0.03). At 6 months, serum albumin in the IDPN group was persistently higher than baseline (*p* = 0.04). Neither volume overload nor uncontrolled hyperglycemia was found throughout the study. In conclusion, a 3-month IDPN supplementation demonstrated a significant increase in serum albumin, body weight, spontaneous oral intake, and MIS; and appeared to be superior to continuing intensive dietary counselling among HD patients intolerable to ONS. The impacts of IDPN therapy on clinical outcomes may require larger scale with longer period of study.

## Introduction

 Protein-energy wasting (PEW), a state of multiple metabolic and nutritional derangements associated with kidney disease, is considered as one of the strongest predictors of death among hemodialysis (HD) patients^1^. The magnitude of PEW is common across the whole spectrum of kidney disease worldwide and its median prevalence reaches 43% in patients undergoing maintenance HD^2^. A cross-sectional report at baseline from the Hemodialysis (HEMO) study revealed that a majority of patients are unable to meet adequate protein and energy intake despite counselling^3^. As a result, the provision of oral nutritional supplements (ONS) is recommended as an appropriate next strategy of nutritional support by the International Society of Renal Nutrition and Metabolism^4^. Although the efficacy of ONS in terms of improving nutritional status in dialysis population has been demonstrated^5,6^, the prolonged period of monotonous supplementation might result in an individual taste fatigue as well as diminished compliance in real clinical practice. Moreover, a previous study reported that approximately 10% of HD patients discontinued ONS because of gastrointestinal complications^7^.

 The National Kidney Foundation Kidney Disease Outcome Quality Initiative (NKF/KDOQI) guideline has nonspecifically suggested a trial option of intradialytic parenteral nutrition (IDPN) for treatment of PEW unless nutritional requirements obtained with existing oral intake or enteral route^8^. IDPN is a form of cyclic parenteral nutrition administered intravenously during each HD session without additional equipment required^9^. In contrast to total parenteral nutrition which is delivered on a daily basis, IDPN is a time-limited modality of nutritional support (usually thrice weekly) while the competency of patient’s gastrointestinal tract function remains unimpaired^10^. In order to maximize the advantageous effects of IDPN as a supplemental nutrition, therefore, the ability of spontaneous protein and energy intake should be achieved to a certain extent. In real clinical practice, the current rate of IDPN use to overcome PEW among dialysis facilities is not uncommon^11^. However, an earlier review of literature has provided inconclusive outcomes regarding the effectiveness of IDPN for treating PEW^12^. In addition, an updated meta-analysis has yielded inconsistent results with respect to the benefits of IDPN among HD patients with PEW, mostly due to unclear selection criteria of participants, lacking of concurrent comparators, and also the heterogeneity of IDPN admixtures^13^.

Indeed, sufficient energy acquisition is necessary to maintain nitrogen balance among HD patients^14^. Therefore, the delivery of IDPN consisting of a combination of three major macronutrients (protein, carbohydrate, and lipid) rather than a single component might provide an improvement of protein-energy status among HD patients with PEW. Apart from uremia-induced malnutrition, the presence of inflammation is also accountable for the imbalance of dietary intake and nutritional targets in patients treated with dialysis^15^. In the present study, we conducted a randomized controlled trial to investigate the effects of IDPN-containing glucose, amino acids, and immune-modulating fish oil-derived lipid emulsion on comprehensive nutritional outcomes that include biochemical and muscle assessments, as well as composite nutritional scoring system such as malnutrition inflammation score (MIS). We also examined the changes of inflammatory- and appetite-related biomarkers after treatment with IDPN.

## Materials and methods

### Study design and participants

This was an open-label randomized controlled study conducted from February through December 2020. All chronic HD patients attending our tertiary care dialysis facility at King Chulalongkorn Memorial Hospital, Bangkok, Thailand and affiliated dialysis centers were screened. Eligible participants were over 18 years of age, receiving maintenance HD for at least 3 months, having spontaneous dietary intake of energy ≥ 20 kilocalorie (kcal)/kilogram (kg)/day and protein intake ≥ 0.8 g/kg/day, being unable to tolerate ONS defined as the inability to tolerate at least one of the adverse gastrointestinal effects such as nausea, vomiting, bloating, or diarrhea, and presence of PEW defined as at least two of the following criteria of serum albumin level ≤ 3.5 g/dL^16,17^, serum prealbumin ≤ 30 mg/dL^18^, mild to moderate malnutrition evaluated by 7-point subjective global assessment (SGA) in category B, or MIS ≥ 5 points^2^. The exclusion criteria were participants with persistent fasting plasma glucose > 300 mg/dL, elevated serum triglyceride > 300 mg/dL, active heart failure, chronic infections such as tuberculosis or human immunodeficiency virus, cirrhosis, metastatic malignancy, pregnancy or lactation, ongoing treatment with immunosuppressive agents or corticosteroid, and history of allergy to any component of parenteral nutrition. All participants had received the standard monthly nutritional counselling in our dialysis center prior to enrollment in this study. This study was approved by the Institutional Review Board of the Faculty of Medicine, Chulalongkorn University (IRB No. 834/62) in compliance with the International guidelines for human research protection as Declaration of Helsinki and International Conference on Harmonization in Good Clinical Practice (ICH-GCP). The study protocol was registered in Thai Clinical Trials Registry (TCTR20191223006) with the full date of first registration on 23/12/2019. All participants provided written informed consent. The study period was 6 months comprising 3 months of intervention and 3 months of intervention-free period. The primary outcome was the change in serum albumin level. The secondary outcomes were the changes of serum prealbumin, muscle mass, strength, composite nutritional scoring system, and biomarkers. From the study of Cano et al.^19^, it was found that IDPN treatment was associated with a significant increase in serum albumin by 0.1 ± 0.2 g/dL in HD patients. To estimate the mean differences of the change of serum albumin of more than 0.22 g/dL with a study power of 80% and α error of 0.05 between two independent samples^20^, the estimated sample size was 15 patients per group. To assume a drop-out rate of 20%, 19 patients were required in each group. A block randomization with equal allocation was used. The allocation was concealed using sequentially numbered, opaque, and sealed envelope.

### IDPN protocol and data collection

All enrolled patients were randomized 1:1 to either receive IDPN plus standard counselling (IDPN group) or intensive dietary counselling (control group) for 3 months. Participants in the IDPN group received the most concentrated 3-in-1 parenteral nutrition formula consisting of glucose, essential and non-essential amino acids and fish oil-based lipid emulsion with omega-3 fatty acids (SMOF Kabiven® central formula 1100 kcal in 986 mL). IDPN was infused at a constant rate during 4 h, but not exceeding 250 ml/hour, via a venous drip chamber of HD machine using infusion pump. The amount of fluid infused was offset by compensated ultrafiltration. The initial delivered volume of IDPN was 8 mL/kg per session and then consecutively up-titrated to achieve the maximal rate of 16 mL/kg per session or the maximal volume of 986 mL according to the European Society for Clinical Nutrition and Metabolism (ESPEN)^21^. Therefore, the highest calorie derived from IDPN was corresponding to approximate 3000 kcal/week. Blood sugar levels were monitored immediately before, 2 h after receiving IDPN, and 30 min after termination of IDPN using glucose test strip. Hyperglycemic events were managed by rapid acting insulin injection administered subcutaneously. Patients allocated into the IDPN group also acquired the monthly individualized nutritional counselling as the standard protocol. In the control group, patients obtained a more frequent intensive dietary counselling by a well-trained renal dietitian once weekly for 3 months. The net calorie and protein deficit was calculated from the actual difference between the recommended macronutrient target and spontaneous oral intake utilizing data from detailed 3-day dietary record including one non-dialysis and one dialysis day as well as a holiday weekend. Patients in the control group were then encouraged to increase their amount of regular food to target total energy and protein intake that complied with the NKF/KDOQI recommendation of 25–35 kcal/kg/day and 1.0–1.2 g/kg/day, respectively^8^. The personalized step diet approach was aimed to augment food portion for main meals or increase number from three to four meals per day followed by adding high-energy snacks between meals. Both groups were then followed for the additional 3 months without intervention until the end of study at 6 months. Any types of ONS were not allowed throughout the study period. Both groups received similar salt restriction (< 5 g/day), exercise duration and intensity, and other standard of cares for HD patients.

### Outcomes measurement

Clinical, biochemical, and body composition analysis by bioelectrical impedance analysis (BIA), and nutritional scoring system using MIS and 7-point SGA were collected at enrollment, 3 months, and the end of 6 months. Blood samples were drawn from blood line before HD at midweek dialysis session. Serum albumin levels were measured using Bromocresol Green method (Alinity c, Abbott, USA). All biochemical tests for nutritional parameters were performed in our central laboratory. The patients’ demographic, comorbidities, current medications, and causes of end-stage kidney disease (ESKD) were extracted from chart review. Study personnel measured height in a standing position using a stadiometer and recorded weight to the nearest 0**.**1 kg using a digital scale**.** Body mass index (BMI) was calculated as post-dialysis weight in kg divided by the square of height in meters**.** We measured blood pressure using the same standard mercury sphygmomanometer after a 15**-**min rest and the average value of 2 consecutive measurements was taken**.** All patients were instructed to regularly collect the 3-day diet record by an experienced renal dietitian and data were carried out using a national nutrition database program (INMUCAL®) based on food composition table for nutritional calculation^22^.

The four compartmental model of BIA (InBody® 720, Seoul, South Korea) was used for muscle mass assessment and made with an eight electrode segmental multi-frequency bioimpedance device using a standard protocol after dialysis for 30 min. The BIA machine was regularly serviced and calibrated. Muscle quality was based on measurement of handgrip strength using a hydraulic hand dynamometer ***(***JAMAR®; Patterson Medical, UK***)*.** We tested handgrip strength on both sides and used the higher of the two. We performed three trials with a 15-s rest period between each trial**.** We discarded the first trial as a “warm up” session, and the highest force exerted in the latter two trials was recorded^22^***.*** For inflammatory markers and appetite-related biomarkers, blood samples were centrifuged at 3000 rpm for 10 to 15 min at room temperature and frozen at − 70 °C for analysis**.** Plasma interleukin-6 (IL-6) and high sensitivity C-reactive protein (hs-CRP) levels were measured by electrochemiluminescence assay. Plasma acylated ghrelin ***(***MyBioSource Inc., CA, USA***)*** as an orexigenic mediator and leptin ***(***Quantikine®, R&D Systems, MN, USA***)***, a surrogate for anorexigenic marker, were measured in duplicate using enzyme-linked immunosorbent assay method and the values averaged.

### Statistical analysis

We described patient characteristics using mean ± standard deviation (SD), or 95***%*** confident interval ***(***CI***) ***for normally distributed or median ***(***25th–75th percentile***)*** for non-normally distributed variables, and proportions for categorical variables. We compared patients’ characteristics using chi-squared, unpaired t-tests, and Mann–Whitney U test as appropriate. The changes in primary and secondary outcomes from baseline in each group were compared using a paired t-test and Wilcoxon Signed-Rank test as appropriate**.** We also used linear regression analyses to test the association of outcomes changes between two groups after treatment and changes of outcomes at 3 and 6 months**.** The data were analyzed in the intention-to-treat analysis**.** We conducted all analyses in Stata 15 ***(***StataCorp LP, College Station, TX***)***, and *p* values less than 0.05 were considered statistically significant^22^.

## Results

### Baseline demographic data of participants

**Figure 1:**
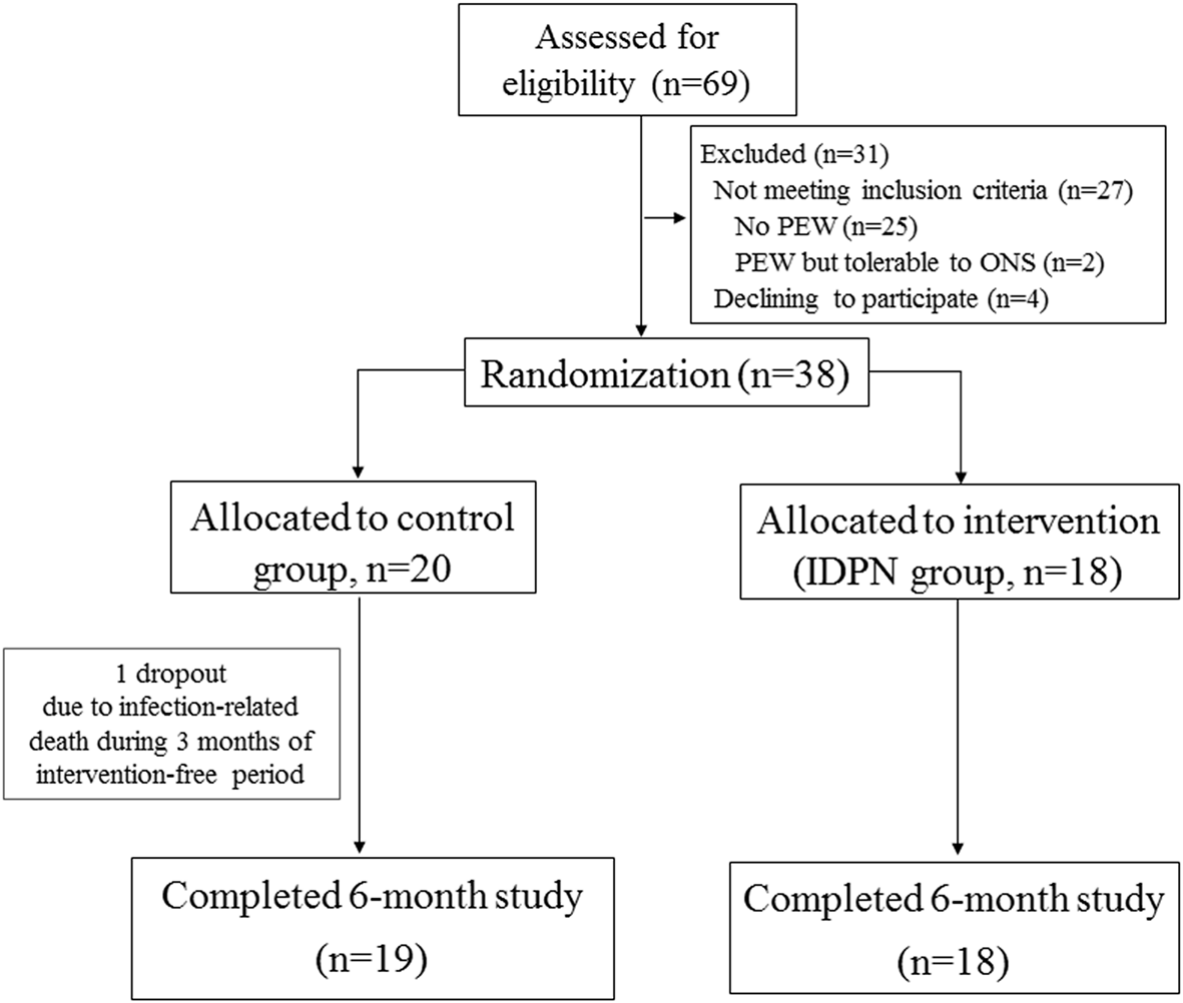
The CONSORT diagram of the study.

Sixty-nine patients were screened for eligibility criteria and a total of 38 patients met the inclusion criteria and were randomized in the study (n = 20 in the control group and n = 18 in the IDPN group). All of the participants completed the 3-month intervention period with one patient dropped out during the intervention-free follow up (Fig. 1). The mean age of the patients was 67.6 ± 10.8 years with 42.1% men. The prevalence of diabetes was 44.7% with the median dialysis vintage of 3.2 (1.2–7.5) years. Approximately 30% of patients had residual kidney function indicated by urine volume > 250 mL/day. All participants met the minimum required target of urea clearance for HD adequacy. The average baseline serum albumin level, spontaneous energy, and protein intake were 3.5 ± 0.3 g/dL, 21.3 ± 7.8 kcal/kg/day, and 0.9 ± 0.3 g/kg/day, respectively. Baseline demographic characteristics including age, sex, dialysis vintage, and laboratory as well as nutritional parameters were not statistically different between 2 groups (Table 1). All patients were either in SGA category A (59.5%) or B (40.5%). Overall, the mean MIS was 8.2 ± 3.2 points with 91.8% of patients had an MIS cutpoint of more than 5, indicating a PEW status.

**Table 1:** Patient characteristics at baseline.

Parameters	Total (n = 38)	Control (n = 20)	IDPN (n = 18)	*p* value
Age, years	67.6 ± 10.8	64.4 ± 11.6	71.1 ± 8.9	0.05
Men, %	42.1	35.0	50.0	0.51
Diabetes, %	44.7	35.0	55.6	0.33
Coronary artery disease , %	31.6	45.0	16.7	0.09
Dialysis vintage, years	3.2 (1.2–7.5)	3.6 (1.5–7.5)	2.2 (1.0–7.5)	0.48
**Cause of ESKD, %**				
Diabetic nephropathy	23.7	10.0	38.9	0.06
Polycystic kidney disease	5.3	5.0	5.6	0.99
Unknown	55.3	70.0	38.9	0.10
**Medication, %**				
Insulin	10.7	10.0	11.8	0.86
ACEI/ARB	27.1	35.0	17.6	0.24
Body weight, kg	57.2 ± 11.6	55.4 ± 11.2	59.3 ± 12.1	0.29
Waist circumference, cm	85.9 ± 9.6	84.0 ± 10.6	88.1 ± 8.2	0.19
Systolic blood pressure, mmHg	147.4 ± 23.4	149.8 ± 22.6	144.7 ± 24.7	0.52
Diastolic blood pressure, mmHg	73.6 ± 11.5	74.1 ± 7.5	73.0 ± 15.1	0.79
Single pool Kt/V urea	1.9 ± 0.4	1.9 ± 0.5	1.8 ± 0.4	0.49
Presence of residual kidney function*, %	28.9	15.0	44.4	0.06
Hemoglobin, g/dL	10.7 ± 1.6	10.5 ± 1.6	10.9 ± 1.5	0.39
Blood urea nitrogen, mg/dL	61.7 ± 18.2	64.3 ± 20.3	58.7 ± 15.5	0.35
Serum creatinine, mg/dL	9.5 ± 3.0	9.9 ± 3.7	9.0 ± 2.1	0.33
Serum bicarbonate, mg/dL	22.3 ± 2.8	21.9 ± 3.0	22.8 ± 2.5	0.34
Serum phosphate, mg/dL	4.6 ± 1.7	4.5 ± 1.7	4.6 ± 1.6	0.73
Serum albumin, g/dL	3.5 ± 0.3	3.6 ± 0.3	3.5 ± 0.3	0.43
Serum prealbumin, mg/dL	27.9 ± 6.8	27.6 ± 7.9	28.2 ± 5.6	0.79
Hemoglobin A1C, %	5.4 ± 1.1	5.2 ± 1.0	5.5 ± 1.1	0.39
Fasting plasma glucose, mg/dL	139.1 ± 57.3	138.0 ± 63.8	140.2 ± 50.9	0.90
Total cholesterol, mg/dL	155.9 ± 46.6	150.9 ± 45.3	161.7 ± 48.8	0.48
Triglyceride, mg/dL	103.5 (77.0–159.0)	112.5 (76.5–163.5)	96.0 (78.0–129.0)	0.56
Low-density lipoprotein, mg/dL	85.5 (66.0–122.0)	79.5 (66.5–104.0)	95.5 (61.0–126.0)	0.73
SGA in category B, %	40.5	31.6	50.0	0.25
MIS, scores	8.2 ± 3.2	7.7 ± 2.8	8.7 ± 3.8	0.39
Normalized PCR, g/kg/day	1.1 ± 0.3	1.1 ± 0.4	1.1 ± 0.3	0.52
**3-day dietary record**				
Energy, kcal/kg/day	21.3 ± 7.8	23.2 ± 7.8	20.0 ± 7.5	0.13
Protein, g/kg/day	0.9 ± 0.3	0.9 ± 0.3	0.8 ± 0.3	0.12

*ACEI* Angiotensin converting enzyme inhibitor, *ARB* Angiotensin receptor blocker, *BMI* Body mass index, *ESKD* End-stage kidney disease, *MIS* Malnutrition inflammation score, *PCR* Protein catabolic rate, *SGA* Subjective global assessment.

Data are presented as mean ± SD and median (25th–75th).

*P* < 0.05 consider significantly different between two groups.

*Residual kidney function defined as urine volume > 250 ml/day.

### Primary outcome and overall protein-energy status

**Figure 2:**
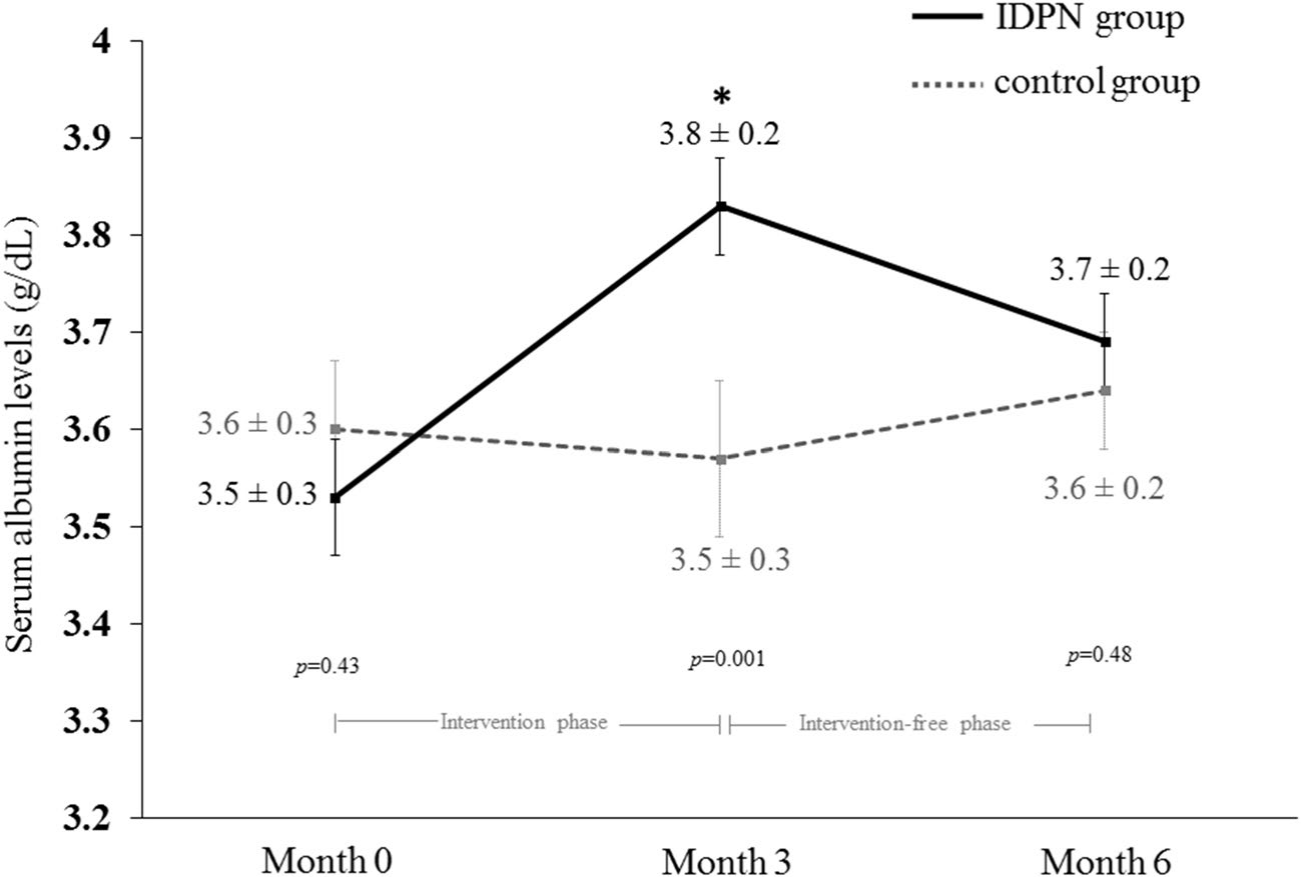
Comparison of serum albumin level as the primary outcome of the study from baseline to the end of study. Data are shown as mean ± SD. *p* values for between groups comparison. *denotes *p* < 0.01 between the control and IDPN group at 3 months.

Among patients receiving IDPN, treatment compliance was 97.3% based on prescribed volume for each dialysis session. There was 3.4% of nonadherence for scheduled weekly administration of IDPN. The mean delivered infusion volume was 14.2 ± 3.9 mL/kg per 4-h HD session which provided the average energy of 902.4 ± 113.3 kcal, protein of 41.0 ± 5.2 g, and omega-3 polyunsaturated fatty acid of 4.5 ± 0.6 g. Total energy intake was increased to 27.6 ± 6.5 kcal/kg/day and total protein intake was raised to 1.1 ± 0.2 g/kg/day after IDPN supplementation. After 3 months of IDPN supplementation, the mean serum albumin level increased by 0.3 (95% CI; 0.2–0.4) g/dL from baseline and was significantly higher in the IDPN compared with the control group (3.8 ± 0.2 vs 3.5 ± 0.3 g/dL, respectively, *p* = 0.01) (Fig. 2). The significant improvement in serum albumin levels did not change after adjusting for age, coronary artery disease, residual kidney function, and body weight (Supplemental Table S1). Body weight was significantly increased from 59.3 ± 12.1 to 61.2 ± 11.9 kg after 3 months of IDPN treatment (*p* = 0.006) whereas it remained unchanged in the control group (from 55.4 ± 11.2 to 56.1 ± 11.4 kg, *p* = 0.22) (Table 2). BMI was also increased in the IDPN group compared with control group after 3 months but this was of borderline statistical significance (*p* = 0.06). After adjusting for baseline consumption, oral energy intake elevated by 281.2 (95% CI; 120.9—441.4, *p* = 0.001) kcal/day and protein intake increased by 8.0 (95% CI; 0.4 – 15.5, *p* = 0.04) g/day at 3 months in the IDPN group. In contrast, spontaneous energy intake was significantly reduced in the control group (from 1,206.8 ± 310.9 to 1,035.2 ± 233.4 kcal/day, *p* = 0.004). Despite IDPN administration for 3-month duration, dialysis adequacy and metabolic profiles in the IDPN were similar to the control group including plasma triglyceride (122.4 ± 57.7 vs 109.9 ± 53.9 mg/dL, *p* = 0.50), hemoglobin A1C (5.5 ± 0.8 vs 5.2 ± 0.7%, *p* = 0.34), pre-dialysis blood urea nitrogen (55.2 ± 15.3 vs 65.7 ± 23.2 mg/dL, *p* = 0.11), and single pool Kt/V urea (2.0 ± 0.4 vs 1.9 ± 0.6, *p* = 0.80) respectively.

**Table 2:** Comparison of changes of primary and secondary outcomes between two groups.

Parameters	Control group (n = 20)		IDPN group (n = 18)		*p* value*
	Baseline	After 3 months	Baseline	After 3 months	
Body weight, kg	55.4 ± 11.2	56.1 ± 11.4	59.3 ± 12.1	61.2 ± 11.9**	0.19
BMI, kg/m^2^	21.9 ± 4.6	22.4 ± 4.4	22.1 ± 3.8	22.6 ± 3.7	0.84
Serum albumin, g/dL	3.6 ± 0.3	3.5 ± 0.3	3.5 ± 0.3	3.8 ± 0.2**	0.001
Serum prealbumin, mg/dL	27.6 ± 7.9	30.0 ± 6.7	28.2 ± 5.5	28.4 ± 6.8	0.46
Energy intake^#^, kcal/kg/day	23.2 ± 7.8	20.0 ± 5.0^†^	20.1 ± 7.5	21.4 ± 5.7**	0.43
Protein intake^#^, g/kg/day	0.9 ± 0.3	0.8 ± 0.3	0.8 ± 0.3	0.9 ± 0.2	0.95
Total-body muscle mass, kg	20.1 ± 3.6	20.2 ± 3.8	20.9 ± 4.9	19.7 ± 3.6	0.69
Appendicular lean mass, kg	14.3 ± 3.3	14.7 ± 3.2	15.1 ± 4.1	14.2 ± 3.3	0.61
Total body fat, kg	17.1 ± 9.8	18.4 ± 9.2	22.1 ± 6.9	22.5 ± 7.2	0.14
Extracellular fluid, L	1.3 ± 1.4	1.3 ± 1.0	1.0 ± 0.7	1.3 ± 1.2	0.98
**Handgrip strength, kg**					
Men	16.3 ± 3.1	20.0 ± 11.3	15.6 ± 6.3	14.4 ± 8.1	0.32
Women	13.1 ± 5.8	11.7 ± 2.7	10.6 ± 3.6	10.8 ± 2.9	0.43
*7-point SGA, %*					
Category A	68.4	88.9	50.0	70.6	0.18
Category B	31.6	11.1	50.0	29.4	0.17
MIS ≥ 5 points, %	89.5	83.3	94.4	76.5	0.61
MIS, points	7.7 ± 2.8	7.0 ± 2.9	8.7 ± 3.8	6.8 ± 3.2**	0.86
Normalized PCR, g/kg/day	1.1 ± 0.4	1.2 ± 0.4	1.1 ± 0.3	1.2 ± 0.2	0.09
Interleukin-6, pg/mL	9.8 (6.8–16.5)	10.4 (6.3–14.1)	7.8 (5.7–18.1)	7.3 (4.7–15.7)	0.35
hs-CRP, mg/L	3.3 (1.8–6.3)	4.0 (1.1–6.5)	3.5 (1.4–8.2)	4.1 (0.9–12.6)	0.95
Acylated ghrelin, pg/mL	160.0 (137.4–178.8)	150.4 (121.4–171.8)	180.8 (134.1–213.6)	161.1 (144.5–187.6)	0.86
Leptin × 10^2^, pg/mL	54.3 (17.5–263.3)	71.9 (32.3–598.4)^†^	141.2 (95.5–311.9)	159.4 (89.9–416.5)	0.88

7-point SGA category A and B defined as well-nourished and mild to moderately malnourished, respectively.

*BMI* Body mass index, *hs-CRP* High sensitivity C-reactive protein, *PCR* Protein catabolic rate. MIS ≥ 5 indicated at risk for protein energy wasting.

^#^Spontaneous energy and protein intake. Data are presented as mean ± SD and median (25th–75th).

*p value between groups comparison at month 3.

**P < 0.01 after 3 months in the IDPN (intervention) group compared with baseline.

^†^*P* < 0.05 after 3 months in the control group compared with baseline.

### Other nutritional-related parameters and biomarkers

**Figure 3:**
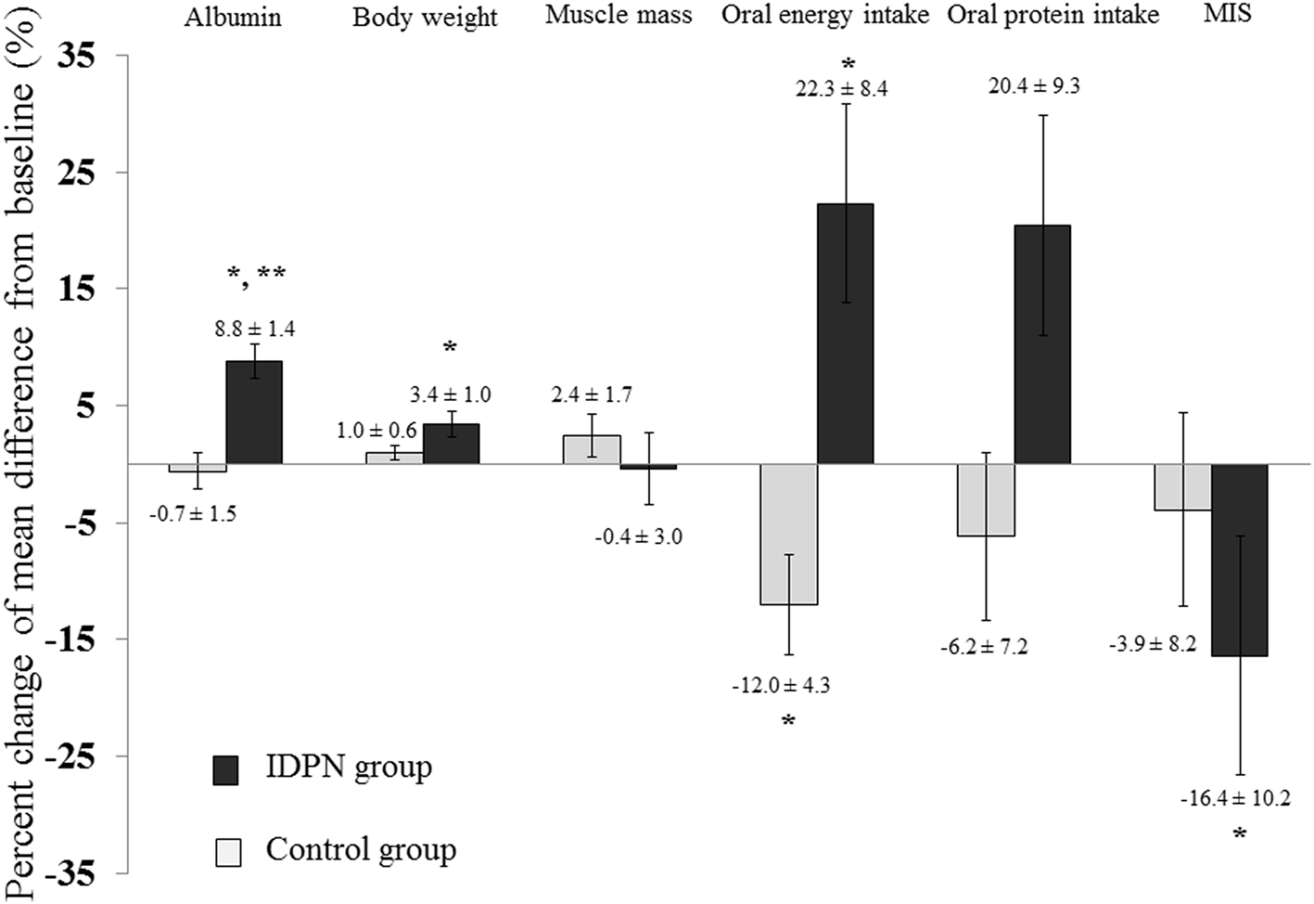
Mean percentage changes of multi-dimensional nutritional assessment according to protein-energy wasting (PEW) criteria including serum albumin as biochemical parameter, body weight as body mass criterion, total-body muscle mass, dietary intake and MIS as an alternative tool after intervention in the IDPN compared with the control group. Data are shown as mean ± standard error of mean (SEM). Percentage change = [(3 months follow up − baseline values)/baseline values] × 100. * denotes *p* < 0.05 after 3 months of IDPN treatment. ** indicates *p* < 0.01 between groups comparison at 3 months.

Serum prealbumin levels were comparable between groups at the end of 3 months. The analysis of body composition revealed that there were no absolute changes of total-body muscle mass (*p* = 0.69), appendicular muscle mass (*p* = 0.61), and total body fat (*p* = 0.14) after 3 months. Muscle function assessed by handgrip strength did not differ between the control and IDPN groups (14.0 ± 7.1 vs 12.5 ± 6.1 kg, respectively, *p* = 0.48) and those results were similar after stratification by gender (Table 2). Although the proportion of patients having MIS ≥ 5 points as categorical value in the IDPN group did not reach statistical significance compared with the control group (76.5 vs 83.3%, respectively, *p* = 0.61), the composite score assessed by MIS as continuous variables significantly reduced from 8.7 ± 3.8 to 6.8 ± 3.2 points after 3 months of IDPN (*p* = 0.005). The non-laboratory criteria of MIS score including patients’ related medical history and physical examination also significantly decreased in the IDPN group (4.9 ± 3.0 to 3.5 ± 2.4, *p* = 0.02). All nutritional outcomes after IDPN treatment was summarized in Fig. 3.

Regarding inflammatory markers, both plasma IL-6 (*p* = 0.35) and hs-CRP levels (*p* = 0.92) did not differ between groups comparison at 3 months. Although plasma IL-6 levels tended to decrease in the IDPN group from the beginning to the end of 3 months, this result did not reach statistical significance (*p* = 0.56) (Table 2). For appetite-related biomarkers, plasma acylated ghrelin levels were not different between groups (*p* = 0.86) and also after at the end of 3 months for each group (*p* > 0.05). Of note, plasma leptin levels were unaltered in the IDPN group after supplementation (*p* = 0.62) whereas it was significantly increased from baseline to 3 months in the control group (*p* = 0.03) (Table 2).

### Changes of outcomes in the intervention-free phase and adverse events

At 6 months, the average serum albumin in the IDPN group decreased by 0.1 (95% CI; -0.3 to 0.4) g/dL but were still significantly higher when compared with baseline (3.7 ± 0.2 vs 3.5 ± 0.3 g/dL, respectively, *p* = 0.04) (Fig. 2). Furthermore, the average spontaneous oral energy and protein intake at 6 months were persistently elevated from the study inception (1,323.3 ± 650.7 vs 1,073.6 ± 408.6 kcal/day, *p* = 0.04 and 53.7 ± 16.1 vs 44.6 ± 17.9 g/day, *p* = 0.02, respectively). In the regression model comparing outcome changes from month 3 to the study exit at month 6, there were no between-group differences in the absolute changes of any other nutritional parameters including body weight (*p* = 0.89), serum prealbumin (*p* = 0.17), and also MIS (*p* = 0.19) (Table 3).

**Table 3:** Changes of nutritional parameters as continuous variables in the IDPN group compared with the control group (between groups comparison).

Parameters	Difference of changes between groups from 0 to 3 months absolute change [95% CI]	*P* value*	Absolute values from month 3 to 6 in the control group (n = 19)	Absolute values from month 3 to 6 in the IDPN group (n = 18)	Difference of changes between groups from 3 to 6 months absolute change [95% CI]	*p* value*
**Primary outcome**						
Serum albumin, g/dL	0.3 [0.2 to 0.4]	< 0.001	3.5 ± 0.3 to 3.6 ± 0.2	3.8 ± 0.2 to 3.7 ± 0.2	− 0.1 [− 0.3 to 0.04]	0.14
**Secondary outcomes**						
Oral energy intake, kcal/kg/day	4.9 [1.6 to 8.3]	0.005	20.0 ± 5.0 to 21.7 ± 8.7	21.4 ± 5.7 to 22.6 ± 8.7	− 2.9 [− 8.7 to 2.9]	0.31
Oral protein intake, g/kg/day	0.2 [− 0.04 to 0.3]	0.13	0.8 ± 0.3 to 0.9 ± 0.3	0.9 ± 0.2 to 0.9 ± 0.2	− 0.2 [− 0.4 to 0.01]	0.06
Serum prealbumin, mg/dL	− 2.3 [− 6.5 to 1.8 ]	0.26	30.0 ± 6.7 to 29.6 ± 7.8	28.4 ± 6.8 to 29.3 ± 7.5	2.1 [− 0.9 to 5.2]	0.17
Body weight, kg	1.4 [− 0.1 to 2.8]	0.06	56.1 ± 11.4 to 56.5 ± 11.3	61.2 ± 11.9 to 60.8 ± 12.2	− 0.1 [− 1.7 to 1.5]	0.89
BMI, kg/ m^2^	0.4 [− 0.3 to 1.0]	0.28	22.4 ± 4.4 to 22.6 ± 4.6	22.6 ± 3.7 to 22.7 ± 3.9	− 0.2 [− 0.8 to 0.3]	0.42
Total-body muscle mass, kg	− 0.8 [− 2.3 to 0.7]	0.27	20.2 ± 3.8 to 20.4 ± 3.5	19.7 ± 3.6 to 20.1 ± 4.5	2.3 [− 1.8 to 6.4]	0.25
Appendicular muscle mass, kg	− 0.4 [− 2.0 to 1.1]	0.56	14.7 ± 3.2 to 14.3 ± 2.9	14.2 ± 3.3 to 14.4 ± 4.0	2.2 [− 1.1 to 5.7]	0.18
Handgrip strength, kg	0.2 [− 4.6 to 5.0]	0.92	14.0 ± 7.0 to 12.0 ± 4.7	12.5 ± 6.1 to 11.6 ± 6.7	1.2 [− 2.9 to 5.2]	0.56
MIS, points	− 1.3 [− 2.9 to 0.4]	0.12	7.0 ± 2.9 to 7.3 ± 3.0	6.8 ± 3.2 to 8.2 ± 4.0	1.0 [− 0.5 to 2.6]	0.19
Normalized PCR, g/kg/day	− 0.1 [− 0.4 to 0.02]	0.08	1.2 ± 0.4 to 1.0 ± 0.3	1.2 ± 0.2 to 1.1 ± 0.3	− 0.01 [− 0.5 to 0.5]	0.98

*BMI* Body mass index, *CI* Confidence interval, *MIS* Malnutrition inflammation score, *PCR* Protein catabolic rate.

*P values for between group comparison of changes using linear regression model.

(−) indicated a decrease value.

No hospital admission was reported in relative to IDPN administration. Neither volume overload requiring hospitalization nor uncontrolled hyperglycemia was found throughout the entire study. The excess extracellular fluid representing overhydration status did not differ between IDPN and the control group (1.3 ± 1.2 vs 1.3 ± 1.0 L, *p* = 0.98, respectively). None of the patients without diabetes and 22.2% of diabetes patients received an additional insulin injection during IDPN infusion. The mean dose of rapid-acting insulin used in the study was 6.0 ± 1.3 (range 4–8) units per dialysis session. At the study completion, there was no statistical significance between groups for other reported adverse events (Table 4).

**Table 4:** Adverse effects of IDPN supplementation in both groups during the entire study period.

Characteristics	Control group (n = 20)	IDPN group (n = 18)^*NS*^
**Major adverse events**		
Infection-related death, n	1	0
Hospitalization due to infection, n	1	1
Non infection-related hospitalization, n	2	4
Volume overload	0	0
Hemorrhagic stroke	1	0
Traumatic bone fracture	0	1
Arteriovenous fistula stenosis	0	1
Elective surgery	1	2
**Minor adverse events**		
Manageable hyperglycemia, n	0	2
Non-specific maculopapular rash, n	0	1
Feeling discomfort, n	0	1

NS, no statistical significant difference between control and intervention group (*p* > 0.05).

## Discussion

The results in the present study demonstrated that a 3-month IDPN supplementation with standard dietary counselling (once-a-month) provided a significant increase in serum albumin compared with continuing intensive dietary counselling (once-a-week) among PEW HD patients intolerable to ONS. Composite nutritional scores assessed by MIS were also significantly improved after IDPN treatment. A significant improvement in spontaneous oral intake after IDPN supplementation was noted and this might be explained, in part, by changes in appetite-related biomarkers.

According to the updated NKF/KDOQI guideline 2020, serum albumin level has been regarded as the most robust predictor of mortality among biochemical indicators for assessment of PEW in HD patients^8^. Data from a large epidemiological cohort revealed that an increase in serum albumin of 0.2–0.3 g/dL was associated with 20% lower likelihood of death after adjusting for covariates^23^. In the present study, we observed that patients receiving IDPN had significantly higher serum albumin in relative to the controls with the average increment by 0.3 g/dL (Fig. 2). In agreement with the current study but having lower number of participants, certain earlier randomized trials^19,24^ showed that serum albumin significantly increased after receiving IDPN when compared with the control arm without IDPN. Previous experimental studies by Pupim and co-workers^25,26^ also supported these observations by illustrating that nutritional support in the form of IDPN enhanced the fractional synthetic rate of albumin in the liver determined by primed constant stable isotope labeled amino acid infusion and increased whole-body protein synthesis. However, the significant improvement of serum albumin level in our study differed from the French Intradialytic Nutrition Evaluation study (FineS)^27^ which revealed that there were no additional benefits of IDPN administration for HD patients with malnutrition. The lack of positive findings in the FineS^27^ might be partly explained by the relatively high rates of IDPN discontinuation (33% at the end of study). Furthermore, the study design used combined interventions of ONS plus IDPN in the investigational arm and compared with ONS in the control arm, resulting in the difficulty to interpret the direct effect of IDPN on outcomes. A recent trial by Marsen and colleagues^28^ indicated that IDPN therapy significantly increased serum prealbumin level but failed to demonstrate a significant change in serum albumin. This disparity in the results might be owing to the lower amount of the infused IDPN volume per session (10.3 ± 4.0 vs 14.2 ± 3.9 mL/kg/session) and higher proportion of severe malnourished participants in their study compared with ours (24.1 vs 0%).

Although prealbumin is often considered as a more sensitive marker due to its shorter half-life than albumin, its serum concentration can be affected by some confounding factors. In recognition that serum prealbumin level is mainly determined by the degradation of retinol-binding protein in renal tubules^29^, this possibility might induce a more interfered result on serum prealbumin concentrations in our study (28.9% having significant residual kidney function) compared with almost anuric ESKD patients enrolled in the study by Marsen et al.^28^.

Preliminary data have suggested that biochemical measurements are insufficiently reliable to use in isolation for assessing nutritional status^30^. In contrast to our report that showed a significant increment in body weight (*p* = 0.01) and a trend toward higher BMI (*p* = 0.06), previous noncontrolled studies^31,32^ found that IDPN therapy using amino acid solution as a solitary component was associated with solely elevated serum albumin. Furthermore, the study by Liu et al.^33^ demonstrated that patients allocated to IDPN using a single glucose solution had a significantly better post-treatment energy status and higher concentrations of plasma essential amino acids profile compared with patients obtaining pure amino acid solution. Given that a majority of maintenance HD patients are facing with calorie depletion without significant amino acid deficiency^34^, we considered that the provision of IDPN consisting of three major macronutrients (glucose, amino acids, and lipid emulsion) might account for the favorable effect of IDPN to replenish energy store, finally leading to improved overall nutritional status as illustrated in our study (Fig. 3). Despite being a non-randomized study design, a very recent work by Demerci et al.^35^ was in agreement with the present study that nutritional status assessed by MIS, a composite nutritional scoring system evaluated by body weight changes over time, BMI, dietary intake, and functional capacity, was also significantly improved after treatment with IDPN mixtures containing completed macronutrients.

The present study is the first to demonstrate that the improvement of spontaneous oral intake after IDPN might be partially mediated by the changes of appetite and inflammatory related biomarkers as part of the proposed pathogenesis of anorexia in PEW HD patients^36^. Although the novel generation of parenteral lipid emulsion extracting from fish oil with omega-3 fatty acid such as eicosapentaenoic acid (EPA) may provide anti-inflammatory properties on immune function^37^, plasma hs-CRP and IL-6 levels were unaffected after 3 months among IDPN and the control groups (Table 2). The lower dose of EPA used in our study (approximately 2820 mg/week) might account for the negative findings compared with a previous report dose of fish oil-related immune modulating effects for at least equivalent to EPA 4200 mg weekly^38^. Our analysis revealed that there was a significant elevation of plasma leptin in the control group whereas it remained unchanged in the IDPN group at 3 months (Table 2). A growing body of evidence indicates that leptin is an adipokine secreted by adipose tissue that exerts inhibitory effects on food intake and is not removed by conventional HD^39^. A previous study among peritoneal dialysis patients showed that the administration of amino acid dialysate was significantly associated with a transient reduction in hyperleptinemia together with increased peritoneal leptin clearance, particularly in the early phase of this non-physiologic mode of nutritional support^40^. However, some earlier studies among chronic HD population indicated that low level of leptin may be inversely associated with high prevalence of PEW and reduced body fat, representing depleted whole-body energy reserve^41,42^. These data suggest that the initial reduction of plasma leptin following nutritional support via IDPN might result in the alleviation of anorexic symptoms whereas the improvement of PEW status after IDPN supplementation might be responsible for a subsequently gradual increase in leptin level that finally lead to the stabilization of serum leptin in our study.

There are scarce data regarding the tolerability and safety of IDPN administration. Even though the large molecular weight of lipid molecules are non-dialyzable and amino acid loads in IDPN mixtures have been formerly reported to reduce dialyzer fractional urea clearance^43^, plasma metabolic profiles and dialysis adequacy were unaltered after IDPN infusion in our study. In spite of a potential of volume overload induced by IDPN, we found no fluid-related complications with the average infusion volume of 14.2 mL/kg per dialysis session in this study. Although fish oil may have more prominent effect on vascular endothelial integrity than other lipid emulsions^44^, the rate of vascular access stenosis was comparable between groups (Table 4). Taken together, a short term IDPN treatment seems to be adverse event-free and somewhat well tolerant among HD patients with PEW.

Certain strengths of this study should be mentioned. We concomitantly performed multi-dimensional nutritional assessment together with relevant biomarkers after IDPN treatment. Moreover, we used all-in-one IDPN admixtures which may be easier to administer than separately compounded solution. The present study also has some limitations to be considered. Albeit appropriate statistical calculation, the number of patients in this study is still quite low. We did not find the significant muscle mass increment although we used multi-frequency BIA instead of anthropometry. Lastly, the effect of increased spontaneous dietary intake is relatively small. Further researches with longer follow up period are crucially needed to establish this beneficial role of IDPN supplementation in improving clinical outcomes among HD population with PEW.

In conclusion, a 3-month IDPN supplementation demonstrated an improvement in serum biochemistry measured by albumin level, body mass status including body weight and BMI, spontaneous oral dietary intake, and also MIS. These beneficial effects of IDPN supplementation appear to be superior to solely extended period of dietary counselling without IDPN among HD patients who could not tolerate to ONS.

## Supplementary Information

Supplementary Information.
