# Supplementary material for: The beneficial effects of intradialytic parenteral nutrition in hemodialysis patients with protein energy wasting: a prospective randomized controlled trial
Source: Sci Rep. 2022 Mar 16;12:4529. doi: 10.1038/s41598-022-08726-8 (PMC8927103; doi:10.1038/s41598-022-08726-8)
Supplement: Supplementary file 1 — Supplementary Information. [file 41598_2022_8726_MOESM1_ESM.docx]

**The Beneficial Effects of Intradialytic Parenteral Nutrition**

**in Hemodialysis Patients with Protein Energy Wasting**

**: A Prospective Randomized Controlled Trial**

Piyawan Kittiskulnam, MD^1,2,3^

Athiphat Banjongjit, MD^2^

Kamonchanok Metta, CDT^2^

Yingyos Avihingsanon, MD^2^

Kearkiat Praditpornsilpa, MD^2^

Kriang Tungsanga, MD^2^

Somchai Eiam-Ong, MD^2,3*^

^1^Division of Internal Medicine-Nephrology, Department of Medicine, Faculty of Medicine,

Chulalongkorn University and King Chulalongkorn Memorial Hospital, Thai Red Cross Society,

Bangkok, Thailand.

^2^Division of Nephrology, Department of Medicine, Faculty of Medicine, Chulalongkorn

University, Bangkok, Thailand

^3^Special Task Force for Activating Research in Renal Nutrition (Renal Nutrition

Research Group), Office of Research Affairs, Chulalongkorn University, Bangkok, Thailand.

Word counts: 4,532

**Short running head**: Intradialytic parenteral nutrition in hemodialysis patients with protein energy wasting

*Corresponding author

Professor Somchai Eiam-Ong, MD

Division of Nephrology, Department of Medicine

Faculty of Medicine, Chulalongkorn University, Bangkok, Thailand 10330

Phone: 662-256-4000, ext. 81014 Fax: 662-252-6929

Email: [somchai80754@yahoo.com](mailto:somchai80754@yahoo.com)

**Supplemental Table S1** Changes of nutritional parameters as continuous variables in the IDPN and the control group (between groups comparison).after 3 months after adjusting for age, coronary artery disease, residual kidney function, and body weight.

| **Parameters** | **Difference of changes**  **between groups**  **from 0 to 3 months** | ***P* value*** |
| --- | --- | --- |
|  | **absolute change [95% CI]** |  |
| *Primary outcome* |  |  |
| Serum albumin, g/dL | 0.3 [0.1 to 0.4] | 0.003 |
| *Secondary outcomes* |  |  |
| Oral energy intake, kcal/kg/day | 3.4 [-0.5 to 7.4] | 0.08 |
| Oral protein intake, g/kg/day | 0.1 [-0.1 to 0.4] | 0.21 |
| Serum prealbumin, mg/dL | -3.5 [-8.3 to 1.3] | 0.14 |
| Body weight, kg | 1.7 [-0.1 to 3.4] | 0.06 |
| BMI, kg/ m^2^ | 0.7 [-0.04 to 1.5] | 0.06 |
| Total-body muscle mass, kg | -0.6 [-2.4 to 1.3] | 0.56 |
| Appendicular muscle mass, kg | 0.2 [-1.6 to 2.1] | 0.78 |
| Handgrip strength, kg | -0.3 [-6.1 to 5.4] | 0.90 |
| MIS, points | -1.3 [-3.4 to 0.7] | 0.19 |

BMI, body mass index; CI, confidence interval; MIS, malnutrition inflammation score

**P* values for between group comparison of changes using linear regression model adjusting for age, coronary artery disease, residual kidney function, and body weight.

(-) indicated a decrease value
